# The effect of modified Qiyuan paste on mice with low immunity and sleep deprivation by regulating GABA nerve and immune system

**DOI:** 10.1186/s13020-024-00939-5

**Published:** 2024-06-12

**Authors:** Mei Rong, Jiu-Jie Jia, Min-Qiu Lin, Xing-Li-Shang He, Zhi-Yi Xie, Ning Wang, Ze-Hua Zhang, Ying-Jie Dong, Wan-Feng Xu, Jia-Hui Huang, Bo Li, Ning-Hua Jiang, Gui-Yuan Lv, Su-Hong Chen

**Affiliations:** 1 Collaborative Innovation Center of Yangtze River Delta Region Green Pharmaceuticals, Zhejiang University of Technology, No. 18, Chaowang Road, Xiacheng District, Hangzhou, 310014 Zhejiang China; 2 College of Pharmaceutical Science, Zhejiang Chinese Medical University, No. 548, Binwen Road, Binjiang District, Hangzhou, 310053 Zhejiang China; 3 Zhejiang Key Laboratory of Innovative Research and Development and Digital Intelligent Manufacturing of Traditional Chinese Medicine Health Products, Huzhou, 310053 Zhejiang China; 4 The Second Affiliated Hospital of Jiaxing University, Jiaxing, 314000 Zhejiang China

**Keywords:** Modified Qiyuan paste (LJC), Low immunity, Sleep deprivation, GABA, Nervous immune system

## Abstract

**Background:**

Low immunity and sleep disorders are prevalent suboptimal health conditions in contemporary populations, which render them susceptible to the infiltration of pathogenic factors. LJC, which has a long history in traditional Chinese medicine for nourishing the Yin and blood and calming the mind, is obtained by modifying Qiyuan paste. *Dendrobium officinale* Kimura et Migo has been shown to improve the immune function in sleep-deprived mice. In this study, based on the traditional Chinese medicine theory, LJC was prepared by adding *D. officinale* Kimura et Migo to Qiyuan paste decoction.

**Methods:**

Indicators of Yin deficiency syndrome, such as back temperature and grip strength, were measured in each group of mice; furthermore, behavioral tests and pentobarbital sodium-induced sleep tests were performed. An automatic biochemical analyzer, enzyme-linked immunosorbent assay kit, and other methods were used to determine routine blood parameters, serum immunoglobulin (IgG, IgA, and IgM), cont (C3, C4), acid phosphatase (ACP) and lactate dehydrogenase (LDH) levels in the spleen, serum hemolysin, and delayed-type hypersensitivity (DTH) levels. In addition, serum levels of γ-aminobutyric acid (GABA) and glutamate (Glu) were detected using high-performance liquid chromatography (HPLC). Hematoxylin–eosin staining and Nissl staining were used to assess the histological alterations in the hypothalamus tissue. Western blot and immunohistochemistry were used to detect the expressions of the GABA pathway proteins GABRA1, GAD, GAT1, and GABAT1 and those of CD^4+^ and CD^8+^ proteins in the thymus and spleen tissues.

**Results:**

The findings indicated that LJC prolonged the sleep duration, improved the pathological changes in the hippocampus, effectively upregulated the GABA content in the serum of mice, downregulated the Glu content and Glu/GABA ratio, enhanced the expressions of GABRA1, GAT1, and GAD, and decreased the expression of GABAT1 to assuage sleep disorders. Importantly, LJC alleviated the damage to the thymus and spleen tissues in the model mice and enhanced the activities of ACP and LDH in the spleen of the immunocompromised mice. Moreover, serum hemolysin levels and serum IgG, IgA, and IgM levels increased after LJC administration, which manifested as increased CD^4+^ content, decreased CD^8+^ content, and enhanced DTH response. In addition, LJC significantly increased the levels of complement C3 and C4, increased the number of white blood cells and lymphocytes, and decreased the percentage of neutrophils in the blood.

**Conclusions:**

LJC can lead to improvements in immunocompromised mice models with insufficient sleep. The underlying mechanism may involve regulation of the GABA/Glu content and the expression levels of GABA metabolism pathway-related proteins in the brain of mice, enhancing their specific and nonspecific immune functions.

**Graphical Abstract:**

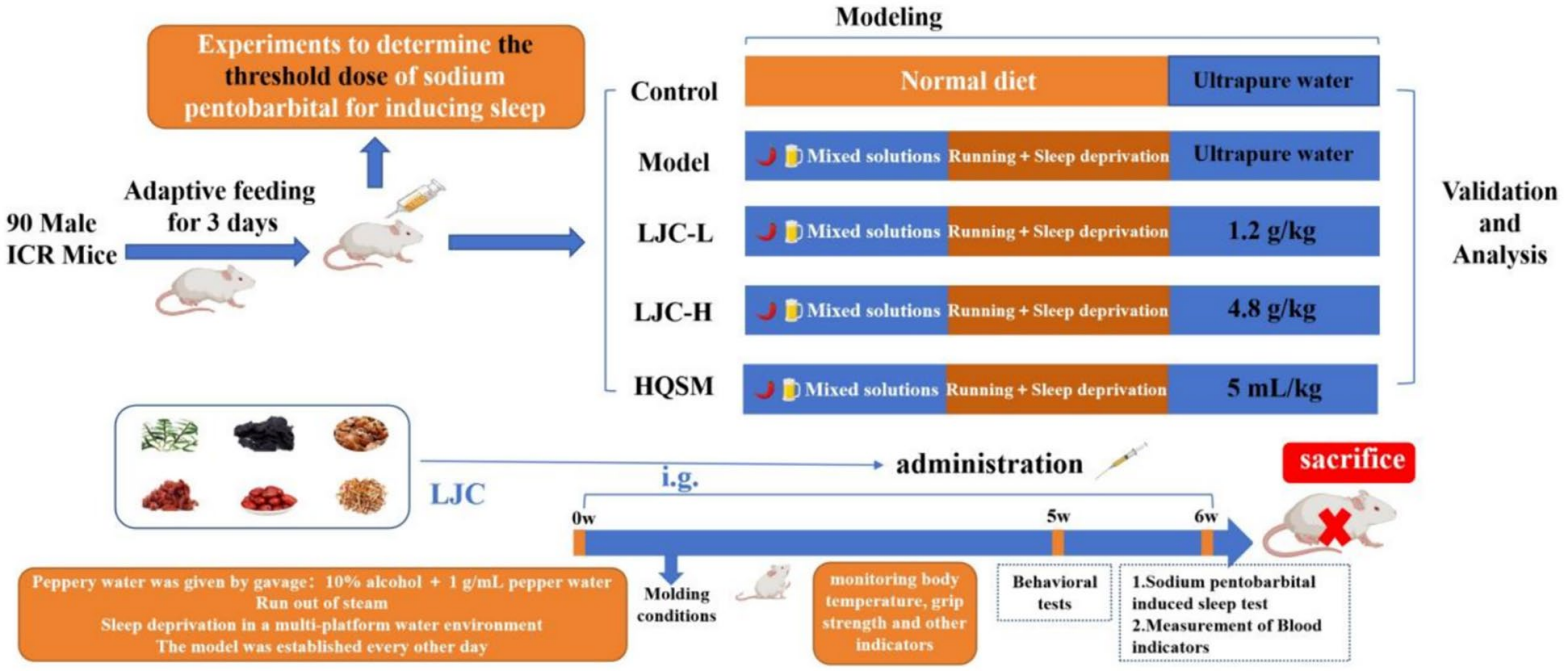

## Introduction

According to modern immunology, immunity is a physiological function of the human body, and the body relies on this function to identify “self” and “non-self” components. This recognition is required to destroy and reject antigenic substances entering the human body or damaged cells and tumor cells generated by the human body itself to maintain physiological balance and health [1–4]. When the immune function declines, the body is in a sub-health state and becomes susceptible to the invasion of pathogenic factors [5–7]. With the rapid advancements in society, there has been a considerable increase in the number of people facing issues such as imbalanced dietary patterns, excessive workloads, elevated physical and mental stress levels, and insomnia. The prevalence of low immunity has become a widespread societal concern and could be attributed to factors such as unhealthy lifestyle habits. According to a survey by the World Health Organization, 75% of the world’s population is in a state of sub-health, and several people suffer from problems such as low immunity and sleep disorders [8]. In recent years, studies have observed that the body’s immune function is closely intertwined with sleep quality [9, 10].

The act of sleeping is a recurring physiological state characterized by physical rest and reduced consciousness, which serves multiple functions, including the enhancement of immune defenses [11]. The human immune system comprises immune organs (bone marrow, thymus, spleen, lymph nodes, etc.), immune cells (lymphocytes, mononuclear phagocytes, neutrophils, basophils, eosinophils, mast cells, platelets, red blood cells, etc.), and immune molecules (complement, immunoglobulin (Ig), cytokines, etc.). In a normal sleep–wake cycle, immune cell numbers, function, and proliferation as well as the production of immune molecules follow their circadian rhythms. For instance, the number of natural killer cells (NK) and neutrophils peaks at noon and reaches its lowest point at night. Mononuclear cells, T-spiral cells (CD^4+^), cytotoxic T cells (CD^8+^), activated T cells (HLA-DR^+^), and B cells (CD19), however, attain their maximum value late at night, then decrease during the rest of the night, and reach their minimum value in the morning [12–15]. Ruiz [16] et al. validated this hypothesis in skin transplanted from mice, showing a redistribution of immune cells during sleep to the spleen and lymph nodes, in contrast to sleep-deprived mice. More recently, a study observed that a sleep restriction of 5 h for 1 week may lead to decreased phagocytosis and NADPH oxidase activity in neutrophils and a reduction in the levels of CD^4+^ T cells [17]. In addition, lack of sleep affects the levels of immune molecules in the body, resulting in alterations in the contents of C3a, IgA, and other molecules [18]. There is growing evidence for the presence of a two-way relationship between sleep deprivation and the immune system: activation of the immune system can affect sleep, and sleep can also have an impact on the immune system [19–23].

Multiple factors modulate sleep and immune responses, and of these factors, GABA and Glu might be two of the main molecules. These are present at extremely high levels in the brain, especially in the hypothalamus, where approximately 30% of the synapses are GABA transmitters [24]. GABA is the major inhibitory neurotransmitter in the central nervous system [25], which is formed by the removal of the carboxyl group by Glu under the action of glutamate decarboxylase (GAD) and is degraded by γ-aminobutyric acid aminotransferase (GABAT). GABA is released from the presynaptic membrane and binds to GABA receptors in the postsynaptic membrane under the action of specific GABA transporters (GATs), thereby exerting inhibitory effects [24, 26]. When its content increases, the duration of slow-wave sleep is prolonged to a certain extent. Studies have shown that GABA transporter subtype1 (GAT1) has a high affinity for GABA and plays a key role in sleep homeostasis [27]. Three types of GABA receptors are present in the human body, namely, GABAA, GABAB, and GABAC. Of these, GABAA is the most abundant and most important receptor in the brain, and it belongs to ligand-gated chloride channel protein [25, 28, 29]. Researchers have shown that there is a decrease in the expression of GABAA receptor α1 subunit (GABRA1) in the hypothalamus of insomnia model animals [30–32]. Glu is a major excitatory neurotransmitter, and low levels of it can cause fatigue and reduce brain activity [33, 34]. Sleep deprivation can cause a series of neurotransmitter-related disorders in the brain, affect the regulation of the sleep–wake cycle, and thus alter the levels of immune cells and immune molecules in the human body [35].

Western medicine mainly relies on providing symptomatic treatment for the occurrence and development of diseases and rarely plays a preventive role. Conversely, traditional Chinese medicine is good at preventing disease and has little toxicity and side effects. Qiyuan paste, derived from the “Secret Anatomy of Health Preservation,” is a time-honored traditional Chinese herbal remedy known for its efficacy in nourishing the Yin and blood, tranquilizing the mind, and enhancing cognitive function. Several studies have documented that *Dimocarpus longan* Lour. and *Lycium barbarum* L*.* can enhance immunity and improve sleep [36–43]. Low immunity and “deficiency syndrome” are similar in various ways according to the concept of Chinese medicine. Traditional Chinese medicine states that *Dendrobium officinale* Kimura et Migo nourishes the Yin and clears the heat, which can effectively improve syndromes related to “Yin deficiency” [44]. In addition, *D. officinale* Kimura et Migo have been reported to considerably improve cellular and humoral immune functions and enhance immunity [45–47]. Therefore, in this study, LJC was prepared by adding *D. officinale* Kimura et Migo to Qiyuan paste based on the theory of traditional Chinese medicine. LJC is a formulation that comprises several traditional Chinese medicines, such as *D. officinale* Kimura et Migo, *Dimocarpus longan* Lour., *Lycium barbarum* L., and *Citrus aurantium* L. This herbal blend nourishes the Yin and promotes circulation; furthermore, it calms the mind and strengthens the spleen. Consequently, it effectively enhances immunity and improves sleep quality.

As per the theory of traditional Chinese medicine and the findings of previous studies, the mouse model of immunocompromised mice with sleep disorders was established by intragastric administration of chili water, sleep deprivation in a multi-platform water environment, and exhausting running to simulate human unhealthy lifestyle of "improper diet, capricious living, and mental loss" [48–51].

Based on previous studies, we hypothesized that LJC may modulate the expressions of GABA signaling pathway proteins in the neuro–immune system and exert an influence on immune organs, immune cells, and immune molecules in the body. This modulation could enhance the immune response, thereby ameliorating sleep disorders and strengthening the overall immunity. Therefore, in this study, a mouse model of low immunity with sleep deprivation was established, and the pharmacodynamics of LJC were evaluated using hematoxylin and eosin (H&E) staining and immunohistochemistry. The contents of immunoglobulin, complement, and GABA/Glu and the expressions of related pathway proteins were determined to clarify the potential mechanism by which LJC enhances immunity and improves sleep.

## Materials and methods

### Chemicals and regents

*Dendrobium officinale* Kimura et Migo. (20,220,928) was purchased from Zhejiang Senyu Co., Ltd (Zhejiang, China). *Dimocarpus longan* Lour. (220,701), *Lycium barbarum* L*.* (220,601), and *Citrus aurantium* L. (20,221,119) was purchased from Zhejiang Chinese Medicine University Chinese Medicine Yinpian Co. Ltd (Zhejiang, China). Huangqi Shengmai decoction (HQSM) (20,220,509) was purchased from Zhejiang Xinguang Pharmaceutical Co., Ltd (Zhejiang, China). hematoxylin–eosin (H&E) dye solution (J22D9Y78310) was purchased from Shanghai Yuanye Biotechnology Co., Ltd (Shanghai, China). Chicken red blood cell (SPF grade) (221,107) was purchased from Guangzhou Hongquan Biotechnology Co., Ltd (Guangzhou, China). Sodium pentobarbital (P258703) purchased from Chengdu Huaxia Chemical Reagent Co., Ltd (Chengdu, China). Acid phosphatase (ACP) test kit (20,221,115), lactate dehydrogenase (LDH) test kit (20,221,107), DAB color development kit (20 ×) (20,221,128) were purchased from Nanjing Jiancheng Bioengineering Institute (Nanjing, China). Toluidine blue (BCBW0650) from Sigma. IgA kit (11/2022), IgM kit (11/2022) and IgG kit (11/2022) were purchased from Shanghai Enzyme Linked Biotechnology Co., Ltd (Shanghai, China). Igg-two-step immunohistochemical kit (17E06D1902) was purchased from Bode Bioengineering Co., Ltd. The complement C3 kit (60,153,325) and complement C4 kit (60,153,370) were purchased from Desai Diagnostic Systems Co., Ltd. L-glutamic acid standard (1112G024), γ-aminobutyric acid standard (1102D022) purchased from Beijing Solaibao Technology Co., Ltd. CD^4+^ primary antibody (HF1116), CD^8+^ primary antibody (HG0806) purchased from Hangzhou Hua 'an Biotechnology Co., Ltd. GAD primary antibody (No.20746–1-AP), GAT1 primary antibody (No.20298–1-AP) and GABRA1 primary antibody (No.12410–1-AP) were purchased from Proteintech Group. GABAT1 primary antibody (YT1819) was purchased from Immunoway.

### Drug preparation

According to the preliminary study, the Chinese herbs in the LJC compound were soaked in distilled water, heated and extracted by reflux. The high concentration LJC extract (crude drug concentration 0.48 g/mL, LJC-H) was prepared by rotary evaporator, and then the high concentration extract was diluted with distilled water to obtain the low concentration LJC extract (0.12 g/mL, LJC-L). HQSM, a positive control in this experiment, was prepared with a final concentration of 0.5 mL/mL suspension, and the daily dosage of mice was 5 mL/kg. The preparation method of pepper water for modeling is to break the dried pepper, extract for 2 h, concentrate the extraction solution, add anhydrous ethanol, adjust the volume to the ethanol concentration of 10%, so that the concentration of the pepper liquid is 1 g/mL containing raw drug.

### The active components of LJC extracts were analyzed by HPLC

The composition analysis of LJC was performed on an Agilent 1260 infinity HPLC system. The analytical column was Welch Ultimate LP C18 (4.6 × 250 mm, 5 µm) at a temperature of 30℃. The flow rate was 1 mL/min, and the injection volume was 10 μL.

Polysaccharide components in LJC: The extract of LJC was stored in cold storage at 4℃ after adding absolute ethanol. After collecting the precipitate, it was put into a vacuum freeze dryer to dry. When removed, crude polysaccharide of LJC extract was obtained. The resulting crude polysaccharide was then prepared by trifluoroacetic acid hydrolysis and PMP derivatization. The mobile phase was 0.025 mol/L potassium dihydrogen phosphate solution **A** and acetonitrile **B**. The detection wavelength was 250 nm. The elution gradient was 0.00–9.00 min with 17%-20%B, 9.01–18.00 min with 20%-22%B, 18.01–26.00 min with 22%-25%B, 26.01–35.00 min with 25%-30%B, 35.01–40.00 min with 30%-37%B, and 40.01–50.00 min with 37%-50%B.

Non-polysaccharide components in LJC: the LJC extract was dried in a vacuum freeze dryer, the lyophilized powder was removed and added to methanol for ultrasound and filtration, then evaporated in a water bath, and then added methanol to redissolve for later use. The detection wavelength was 210 nm. The elution gradient was 0.00–20.00 min with 5%-10%B, 20.01–30.00 min with 10%-15%B, 30.01–35.00 min with 15%-18%B, 35.01–40.00 min with 18%-22%B, 40.01–46.00 min with 22%-28%B, 46.01–50.00 min with 28%-30%B, 50.01–60.00 min with 30%-35%B, 60.01–75.00 min with 35%-55%B, and 75.01–80.00 min with 55%-65%B.

Preparation of Reference Substances: The glucose, rhamnose, arabinose, mannose, galactose, glucuronic acid, glucosamine, ribose and xylose reference materials were weighed carefully, and methanol was added to make a solution of 1 mg/mL. Precision weighing vitenine-1, vitenine-2, naringenin, naringin, rutin, chlorogenic acid, quercetin, luteolin, hesperidin control substance amount, precision weighing, adding methanol to make a solution of 1 mg/mL.

### Animals and treatment

ICR male mice weighting 20 ± 2 g were obtained from Hangzhou Qizhen experimental animal Technology Co., Ltd. The animals (SCXK (Zhe)2022–0007) were maintained at a constant room temperature of 22–26℃ and a humidity of 50–70% for 12 h light/dark cycles. They were adaptively given food and water for 3 days. All experiments were performed complied with the Regulation of Experiment Animal Administration issued by the Ministry of Science and Technology of the People’s Republic of China. The experiment was received approval by ethics committee of Zhejiang University of Technology (20221125Abzz0100999262).

Before the experiment, 90 male ICR mice were randomly divided into 6 groups (n = 15) with sodium pentobarbital dose of 55, 50, 45, 40, 35, and 30 mg/kg, respectively. After intraperitoneal injection of the corresponding dose of sodium pentobarbital in each group, Sleep latency time of mice within 20 min (from the time of injection of pentobarbital sodium to the time of disappearance of righting reflex) and the duration of sleep of mice (from the time of disappearance of righting reflex to recovery) were recorded, and the dose of 100% sleep of mice without too long sleep time was determined as the above threshold dose of pentobarbital sodium. The subthreshold dose of pentobarbital sodium in 80–90% mice whose righting reflex does not disappear is subthreshold dose.

Then 50 male ICR mice were randomly divided into 5 groups (n = 10). They were divided into control group, model group, LJC-H group (4.8 g/kg, LJC high dose), LJC-L group (1.2 g/kg, LJC low dose), and HQSM group (5 mL/kg). In this experiment, the model was established while the drug was given for 6 weeks. The modeling method is as follows: In addition to the control group, the other groups were given gavage to make thermotropic drugs in odd days (the dose for the first two weeks was 1.6 g/kg, and the adjusted dose was 5 g/kg later), and underwent exhaustive running training (the adaptive running training was conducted for one week, and the final running time was 30 min and the running speed was 25 m/min). Even days, sleep deprivation was performed in a multi-platform water environment (12 h of sleep deprivation for the first two weeks, then adjusted to 18–21 h). The control group and model group were given the corresponding volume of normal temperature water by intragastric administration, and the other groups were given the corresponding therapeutic drugs by intragastric administration of 0.1 mL/10 g, once a day, for 6 weeks. Then, blood was collected through the orbit and after the mice were sacrificed, the brain tissue, spleen tissue and thymus tissue were collected and stored in the refrigerator at − 80℃.

### Indicators of “Yin deficiency” syndrome

The dorsal region of the mice was captured using a thermal camera, and the temperature was subsequently calculated utilizing FLIR ONE software. Rectal thermometry was employed to measure and record the anal temperature of the mice. Additionally, each mouse underwent a grip strength test where it was pulled back at a predetermined speed until releasing its claw, allowing for recording of maximum grip force. Saliva flow rate in mice was assessed by cutting filter paper into small pieces which were then inserted into their mouths using tweezers. After 5 s, the dry weight and wet weight of the filter paper were measured, enabling calculation of saliva flow rate as an increase in weight per second.

### Pentobarbital sodium-induced sleep tests

The pentobarbital sodium-induced sleep test in mice is a commonly used behavioral method to assess whether a drug has sedative-hypnotic activity [52]. A pentobarbital sodium induced sleep test was performed 2 days before the end of administration. 1 h after the gavage, the mice were intraperitoneal injection of pentobarbital sodium (the upper threshold dose was determined to be 50 mg/kg in the pre-experiment), and the sleep duration of mice was immediately recorded.

### Behavioral test

Autonomous activity experiment in the 5th week of the experiment [53]. 30 min after administration, the mice of each group were placed into multifunctional mouse automatic activity recorder to acclimate for 2 min, and then the locomotion activity of each mice was measured and collected within 5 min.

Elevated plus maze (EPM) was performed on mice in the 5th week of the experiment [54]. The labyrinth is composed of two open arms and two closed arms. The arm length is 25 cm, and the central area is a square grid of 5 × 5 cm. The EPM is elevated 40 cm above the floor. During the test, the mice were placed face to face into the central area of the open arm, and the activities of the mice were filmed with a camera for 5 min. The percentage of time and the times of the mice entering the open arm were counted in the later period.

Open-field test (OFT) in the 5th week of the experiment [55]. After the drug administration for 30 min, mice in each group were subjected to an open-field test. The open field is composed of 40 × 40 × 40 cm cartons, and the bottom form is 5 × 5 lattice. During the test, the mice were placed in the center of the open field box, and the activities of the mice for 5 min were recorded by the camera, and the number of cells passed by the mice (recorded as horizontal scores) and the number of times of standing (recorded as vertical scores when the two front feet were lifted or attached to the wall of the box) were counted. The software EthoVision XT17 was used to calculate the moving distance and moving speed, and the track chart of the mice's open field activities was drawn.

### The detection of blood indexes and other immune indicators

The level of hemolysin production and delayed hypersensitivity (DTH) can reflect the immunomodulatory ability of the body [56]. The mice in each group were intraperitoneally injected with 5% chicken red blood cells for 4 consecutive days, with an injection dose of 0.2 mL per mouse. 1 h after the final administration, orbital blood was collected and the supernatant was obtained through centrifugation. After centrifugation, 10 μL of serum was taken and diluted 100 times with normal saline. Then, 0.5 mL of 5% chicken red blood cells and 0.5 mL of fresh guinea pig serum (10%) were added to the diluted serum. After mixing, the reaction was stopped by incubating at a water bath temperature of 37℃ for 60 min followed by an ice bath treatment. Subsequently, centrifugation (2000 r/min, 10 min) was performed to separate the supernatant from the mixture. In the blank control group, normal saline was used as a replacement for serum samples. The absorbance at a wavelength of 540 nm was measured.

Additionally, after 4 days, the initial thickness of the left foot was measured using a vernier caliper three times on average. Subcutaneous injection of 50% chicken red blood cells (20 μL) took place at the same measurement site. After a period of 24 h had passed since injection, another measurement using a vernier caliper 3 times on average determined the thickness change in the left foot as DTH response data.

At week 6 of dosing, mice in each group were water deprived for 12 h, and orbital blood was collected through EDTA anticoagulant tubes. The white blood cell count and the proportion of white blood cells in the blood of mice were determined by automatic hematology analyzer.

Before the end of the experiment, the blood was collected from the orbit of mice and incubated in Ep tube at 37℃ for 30 min. The blood was centrifuged twice (3600 r/min, 10 min) and the upper serum was collected. Enzyme-linked immunosorbent assay (ELISA) was used to detect the content of immunoglobulin (IgG, IgA, and IgM) in serum according to the instructions. The levels of complement (C3 and C4) in serum of mice were detected by automatic biochemical analyzer.

ACP and LDH are important indicators of macrophage function and their high presence in macrophages suggests it plays a vital role in immunology [57–59]. On the other hand, GABA has been found to activate macrophages and promote their proliferation, autophagy and secretory functions [60]. Therefore, at the end of the experiment, the spleens of mice was weighed, 9 times the volume of normal saline was added, and ground by a high-throughput tissue grinder. After centrifugation (3500 r/min, 10 min), the supernatant was taken for determination of the protein concentration in the spleen. The contents of ACP and LDH in the spleen were detected according to the operating steps of the kit.

### Histological evaluations

H&E staining was used for the histopathological examination of the thymus and spleen, and the histopathological examination of hypothalamus tissue was evaluated by H&E and Nissl staining. Thymus, spleen and brain tissues were fixed with 4% formalin solution, dehydrated in different concentration of alcohol, and embedded in paraffin. After that, they were cut into 4 µm paraffin section and stained by H&E [61]. At the same time, the number of Nissl bodies was determined by Nissl staining. Paraffin embedded hippocampus sections were cut into 4 µm sections, and then the sections were stained with toluidine blue water solution at 50–60℃ for 10 min to observed Nissl bodies in nervous cells [62].

### HPLC analysis of γ-GABA and Glu

Same as the previous study [63]. Firstly, 0.5 mL of perchloric acid (0.4 mol/L) was added to 20 μL serum (diluted 5 ×) and centrifuged at 3000 rpm/min for 15 min to remove the protein. Then, 50 μL supernatant was added to 125 μL Na_2_CO_3_ (1 mol/L) to prepare the sample for HPLC detection. The derivatization reaction was carried out with 200 μL of sample and 400 μL of o-phthalaldehyde (OPA) (5 mg OPA was dissolved in 100 μL of methanol solution, 5 μL of 2-mercaptoethanol was added, and diluted to 5 mL with borate and 0.4 mol/L sodium hydroxide buffer). The contents of amino acids γ-GABA and Glu were determined by HPLC. An EC-C18 column (4.6 mm × 150 mm, 4 µm) was used as the injection column at 30℃ with acetonitrile–water (50:50, A) and sodium acetate (0.05 mol/L) as the mobile phase. Variable wavelength scanning UV detector (VWD) wavelength was set at 338 nm. The mobile phase A increased by 2% per minute, and the end was 30 min.

### Immunohistochemistry and Western Blot Assay

The expression of GABRA1, GAD, GAT1, and GABAT1 proteins in the brain was evaluated by immunohistochemical staining. The brain paraffin tissue sections were incubated with GABRA1, GAD, GAT1, and GABAT1 antibodies, then the sections were incubated with secondary antibody goat anti-rabbit IgG, the signals were observed by DAB solution and the nuclei were counterstained with hematoxylin. At the same time, the expression of CD^4+^ and CD^8+^ protein in thymus and spleen tissues was detected by the same method. The positive expression showed yellow color under microscope. The results of protein levels were evaluated by detecting the integrated option density (IOD) in positive area [64].

Western blot was used to detect the expression of GABA, GAD, and GAT1 protein. Brain protein was extracted with radioimmunoprecipitation (RIPA) buffer, and protein concentration was determined by BCA protein assay kit. The total protein (100 μg) was separated by sodium dodecyl sulfate polyacrylamide gel electrophoresis (SDS-PAGE) and transferred to polyvinylidene fluoride (PVDF) membrane. After blocking for 2 h, the protein was incubated with primary antibody at 4℃ overnight and washed with TBST. Horseradish peroxidase conjugated affinity purified goat anti-rabbit IgG (H + L) secondary antibody (1:10,000) was added and incubated with the target protein. The results were visualized by chemiluminescence (ECL) Western blot detection system. Image J image analysis software was used to analyze the optical density value of the bands, and the expression level was standardized by the relative expression of the target protein (optical density value of the target protein/optical density value of the internal reference protein β-actin). And then the expression level of the protein was analyzed.

### Statistical analysis

All results were presented as means ± standard deviation (SD). Results were statistically evaluated using IBM SPSS Statistics 19.0. Significant differences between groups were determined by a Student's t-test or one-way analysis of variance (ANOVA), the graphs were performed by GraphPad Prism 8.0.

## Results

### Active component analysis of LJC

**Fig. 1:**
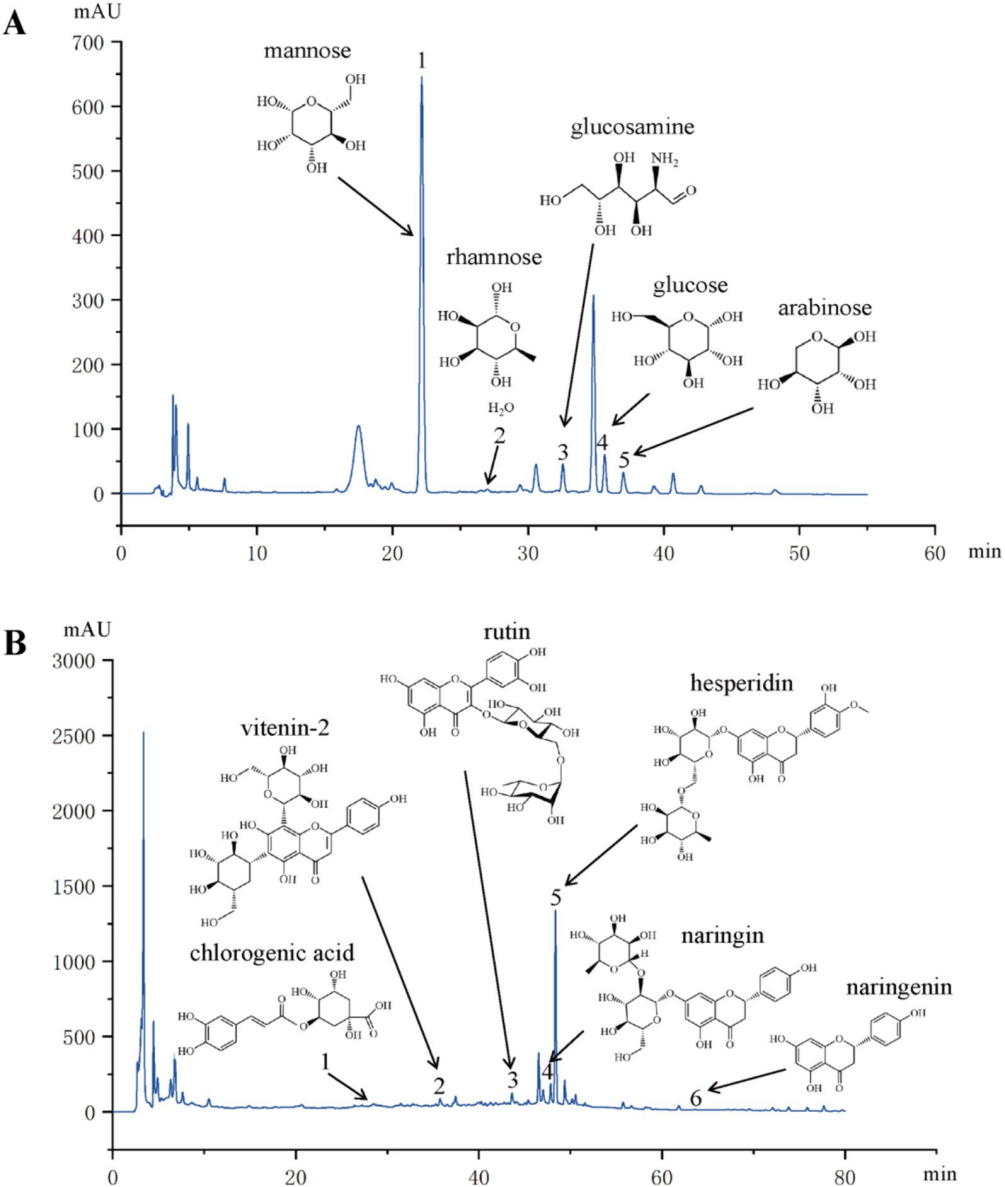
The HPLC profile of the active constituents in LJC. **A** HPLC plot of polysaccharide, 1: mannose, 2: rhamnose, 3: glucosamine, 4: glucose, 5: arabinose; **B** Non-polysaccharide HPLC plot, 1: chlorogenic acid, 2: vetsenin-2, 3: rutin, 4: naringin, 5: hesperidin, 6: naringenin

Our preliminary experiments determined that the main composition of LJC polysaccharide were mannose, rhamnose, glucosamine, glucose and arabinose, and the non-polysaccharide component were mainly chlorogenic acid, vitenin-2, rutin, naringin, hesperidin and naringenin. The typical chromatograms are presented in Fig. 1.

### Changes in body temperature, grip, and saliva flow rate treatment with LJC

In order to evaluate the effect of LJC on Yin deficiency syndrome in mice with low immunity and sleep deficiency, the changes of back temperature, anal temperature, grasping power and saliva flow rate of mice in each group were collected.

**Fig. 2:**
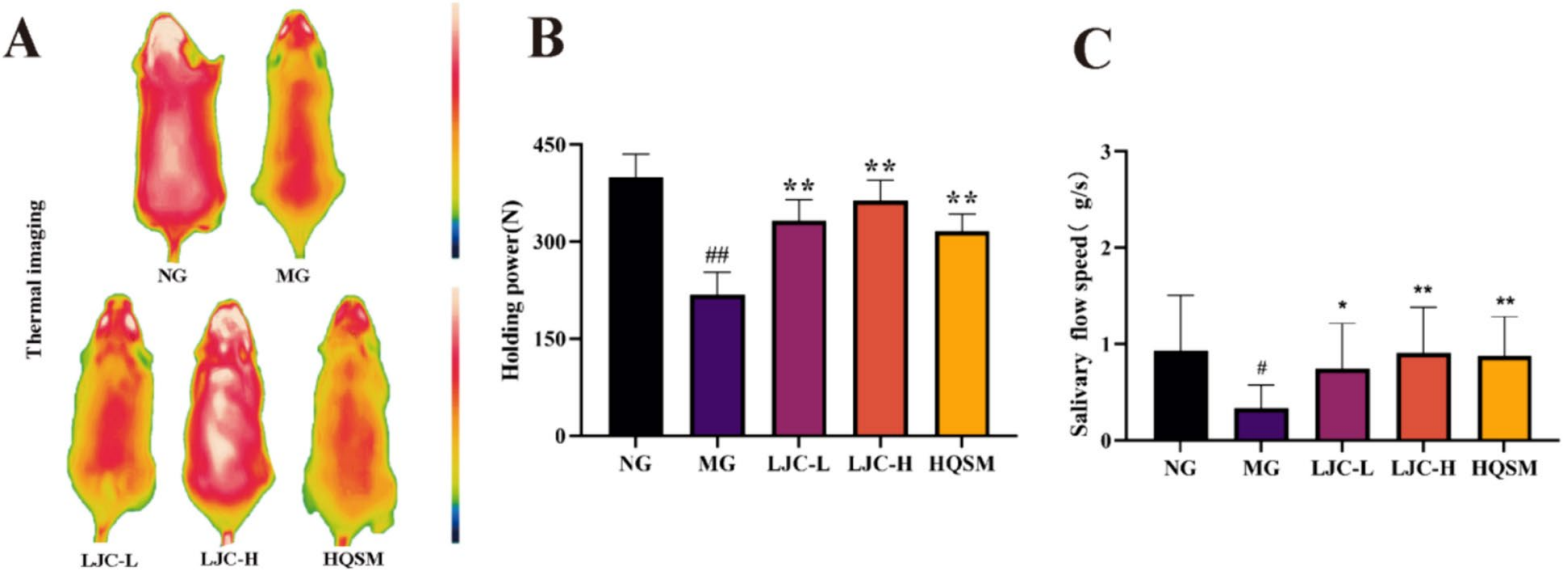
Effects of LJC treatment on body temperature, grip strength, and salivary flow rate. **A** Thermal imaging; **B** Holding power; **C** Salivary flow speed. Data are expressed as the mean ± SD. ^#^*P* < 0.05, ^##^*P* < 0.01 compare with the control group; ^*^P < 0.05 and ^**^P < 0.01 compare with the model group

Compared to the normal group, mice in the model group gradually exhibited symptoms of "Yin deficiency" after modeling, including increased body temperature, fatigue, and dry mouth. These symptoms were significantly improved in the medication group. Figure 2A and Table 1 shows the changes of back and anal temperature in mice. After 2 weeks of modeling, compared to the model group, each treatment group showed a significant decrease in back temperature (*P* < 0.01). After 4 weeks of modeling, compared to the model group, only the LJC group demonstrated a significant reduction in rectal temperature (*P* < 0.01). This suggested that LJC can effectively alleviate symptoms related to Yin deficiency and internal heat in modeled mice.

**Table 1:** Effects of LJC on back temperature and rectal temperature in mice

Group	Back temperature at week 2 (℃)	Rectal temperature at week 4 (℃)
NG	28.97 ± 0.90	36.06 ± 0.59
MG	29.73 ± 0.44^#^	37.07 ± 0.34^##^
LJC-L	28.98 ± 0.64^**^	36.49 ± 0.54^**^
LJC-H	28.97 ± 0.69^**^	36.38 ± 0.71^**^
HQSM	28.69 ± 0.91^**^	36.68 ± 0.65

*#P* < 0.05, *##P* < 0.01 compare with the control group; *P < 0.05 and **P < 0.01 compare with the model group

Figure 2B demonstrated that holding power significantly recovered after LJC and HQSM treatment (*P* < 0.01), with a higher degree of improvement observed in the LJC group than in the HQSM group. This indicated that LJC can effectively improve symptoms associated with Yin deficiency fatigue in modeled mice.

The salivary flow speed index was used to quantify dry mouth symptoms in mice. As shown in Fig. 2C, all treatment groups exhibited a significant increase in salivary flow speed compared to the model group (*P* < 0.05, 0.01). This suggested that LJC can effectively alleviate dry mouth symptoms in modeled mice.

### Sedative effect of LJC on insomnia Mice

**Fig. 3:**
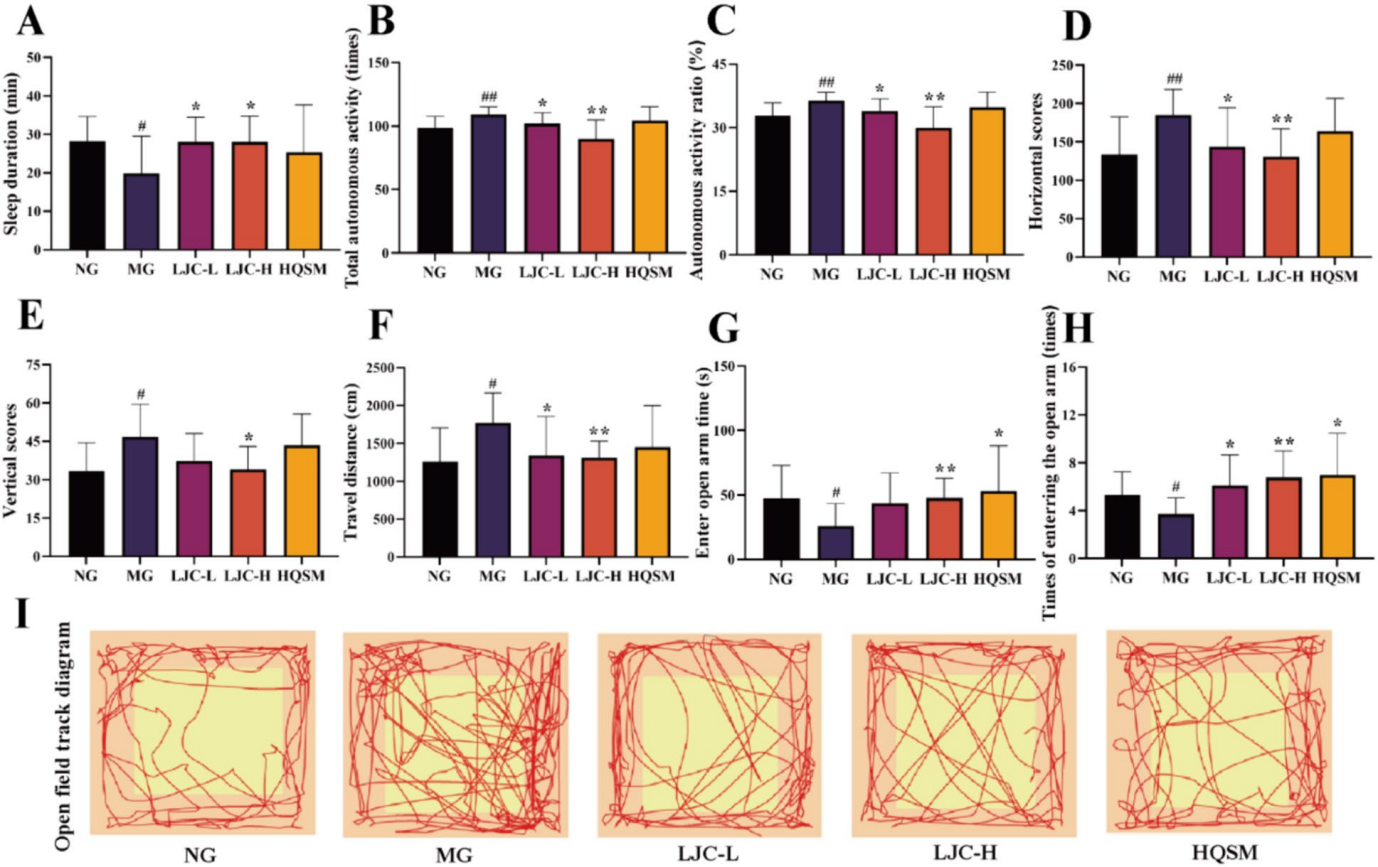
Sedative and hypnotizing effects of LJC in model mice. **A** Sleep duration; **B** Total autonomous activity; **C** Autonomous activity ratio; **D** Horizontal scores; **E** Vertical scores; **F** Travel distance; **G** Enter open arm time; **H** Times of enterring the open arm; **I** Open field track diagram. Data are expressed as the mean ± SD. ^#^*P* < 0.05, ^##^*P* < 0.01 compare with the control group; ^*^P < 0.05 and ^**^P < 0.01 compare with the model group

The effect of LJC on sleep duration induced by a hypnotic dose of sodium pentobarbital (50 mg/kg) was shown in Fig. 3A after hypnotic dose of sodium pentobarbital mice. Compared with the control group, the sleep duration of the sleep-deprived mice in the model group was significantly shortened (*P* < 0.05). Compared with the model group, the LJC group had a significant increase in sleep duration (*P* < 0.05) significantly enhanced the hypnotic effect of sodium pentobarbital.

The effects of LJC on anxiety and exploratory behavior of sleep deprived mice were evaluated using autonomic activity test, elevated plus maze test, and open field test. The model group exhibited significantly higher autonomic activity compared to the control group (*P* < 0.01). However, the LJC group showed a significant decrease in autonomic activity (*P* < 0.05, 0.01) (Fig. 3B, C). In the open field test, the model group mice demonstrated significantly increased transverse and longitudinal scores as well as moving distance compared to the control group (*P* < 0.05, 0.01) (Fig. 3D–F). On the other hand, compared to the model group, both lateral movement scores and moving distance were significantly reduced in the LJC group (*P* < 0.05, 0.01), while only longitudinal scores were decreased in the LJC-H group (*P* < 0.05). As shown in Fig. 3G, H, the time spent entering into open arms as well as number of entries into open arms were significantly reduced for mice in model groups when compared with those from normal groups (*P* < 0.05). Conversely, both time spent entering into open arms as well as number of entries into open arms for mice treated with LJC-H were markedly increased when comparing with those from model groups (*P* < 0.01). Notably, when comparing with the normal group, irregular and disorganized activity trajectories were observed in the model group; however after treatment with LJC and HQSM, mice in each experimental groups displayed stable and regular activity trajectories similar to those seen in normal mice (Fig. 3I). Therefore, we concluded that LJC exerted favorable sedative effect on sleep-deprived mouse models.

### The effects of LJC on immune cells and immune molecules

**Fig. 4:**
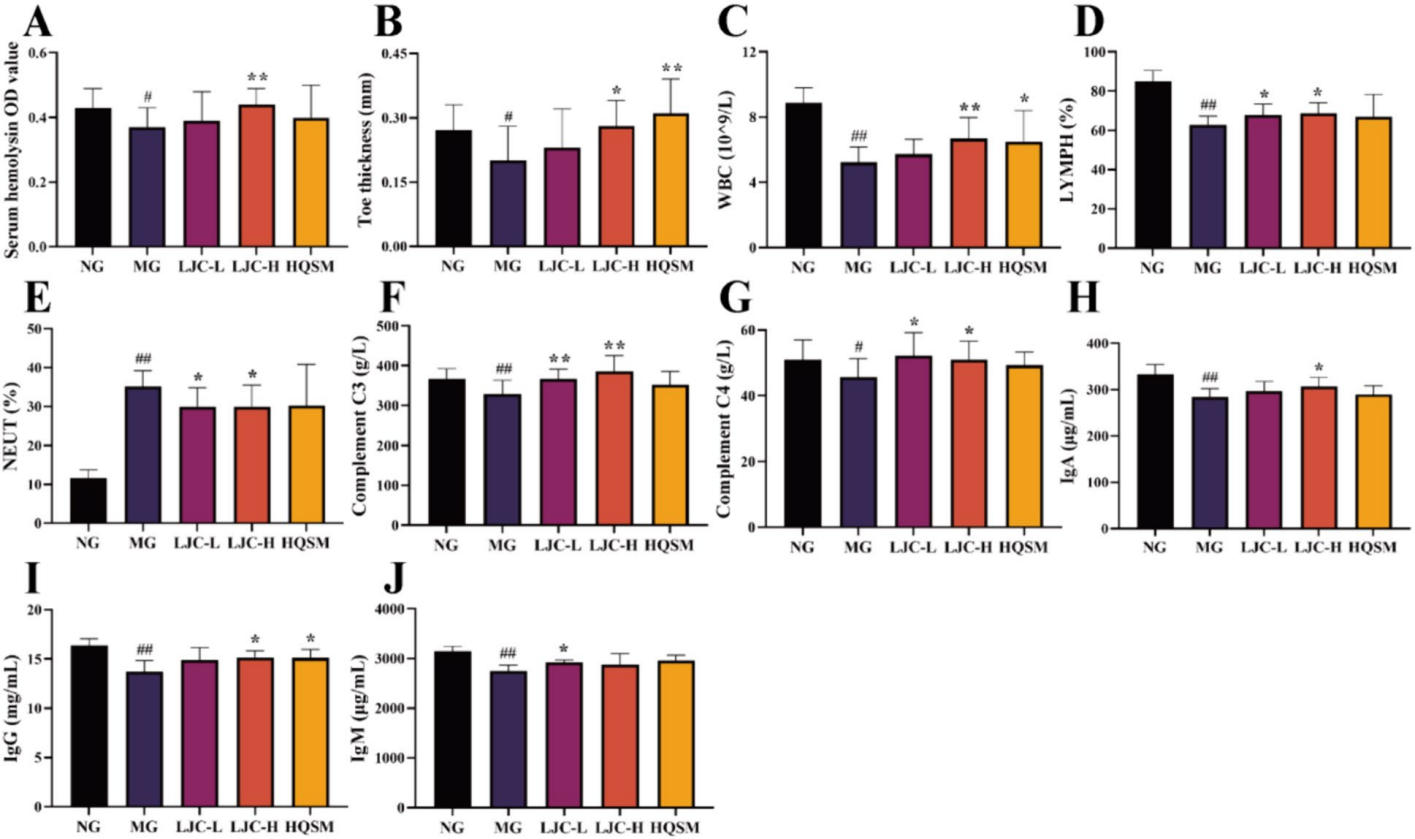
Effects of LJC on immune cells and immune molecules in the blood. **A** Serum hemolysin levels; **B** Toe thickness; **C** Peripheral white blood cell count; **D** The percentage of peripheral blood lymphocytes; **E** The percentage of neutrophils in peripheral blood; **F** Serum complement C3 level; **G** Serum complement C4 level; **H** Serum IgA level; **I** Serum IgG levels; (J) Serum IgM levels. Data are expressed as the mean ± SD. ^#^*P* < 0.05, ^##^*P* < 0.01 compare with the control group; ^*^P < 0.05 and ^**^P < 0.01 compare with the model group

As shown in Fig. 4A, B, after 4 weeks of treatment, compared with the normal group, the serum hemolysin level and paw swelling degree of the model group mice were significantly decreased (*P* < 0.05); Compared with the model group, the serum hemolysin level of the LJC-H group was significantly increased (*P* < 0.01). In contrast, the paw swelling degree of LJC-H and HQSM groups increased significantly (*P* < 0.05, 0.01). It was suggested that LJC could increase serum hemolysin level and promote DTH reaction.

After 5 weeks of treatment, the number of white blood cells and the percentage of lymphocytes in the peripheral blood of the mice in the model group were significantly lower than those in the normal group (*P* < 0.01), while the percentage of neutrophils was significantly increased (*P* < 0.01); Compared with the model group, the number of white blood cells in the LJC-H group increased significantly (*P* < 0.01). In terms of lymphocyte percentage, both LJC-L and LJC-H groups had varying degrees of increase (*P* < 0.05), and the percentage of neutrophils in LJC-L and LJC-H groups decreased significantly (*P* < 0.05) (Fig. 4C–E). These results suggested that LJC could significantly increase the number of white blood cells in immunocompromised mice with sleep disorders.

Compared with the normal group, the levels of serum complement C3 and C4 in the model group were significantly decreased (*P* < 0.05, 0.01); Compared with the model group, the levels of C3 and C4 in the LJC treatment group were increased to varying degrees (*P* < 0.05, 0.01) (Fig. 4F, G). These results suggest that LJC may enhance immunity by activating the complement system.

After 6 weeks of modeling, compared with the normal control group, the serum levels of IgA, IgG and IgM in the model control group were significantly decreased (*P* < 0.01), the levels of IgA, IgG, and IgM were increased in different doses of LJC groups (*P* < 0.05, 0.01). It was suggested that LJC could improve the immunity by stimulating B lymphocytes to secrete antibodies. The results are shown in Fig. 4H–J.

### Histopathological evaluation of brain tissue

**Fig. 5:**
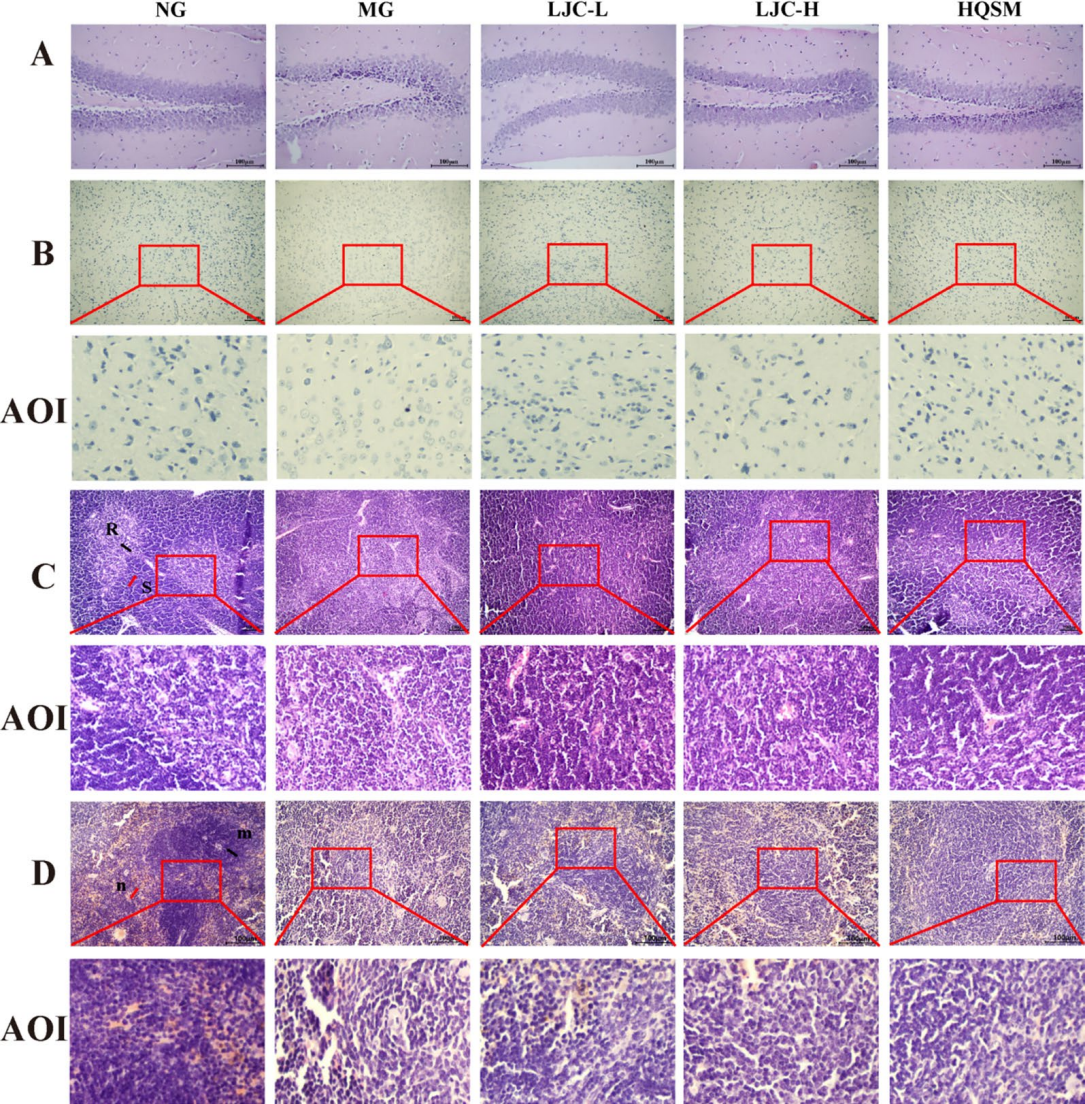
Histopathological evaluation of brain tissue, thymus and spleen. **A** H&E staining of hippocampus; **B** Nissant staining; **C** Thymus H&E staining (200 ×), R: thymus medulla, S: thymus cortex; **D** H&E staining of spleen (400 ×), m: white pulp area of spleen, n: red pulp area of spleen

**Fig. 6:**
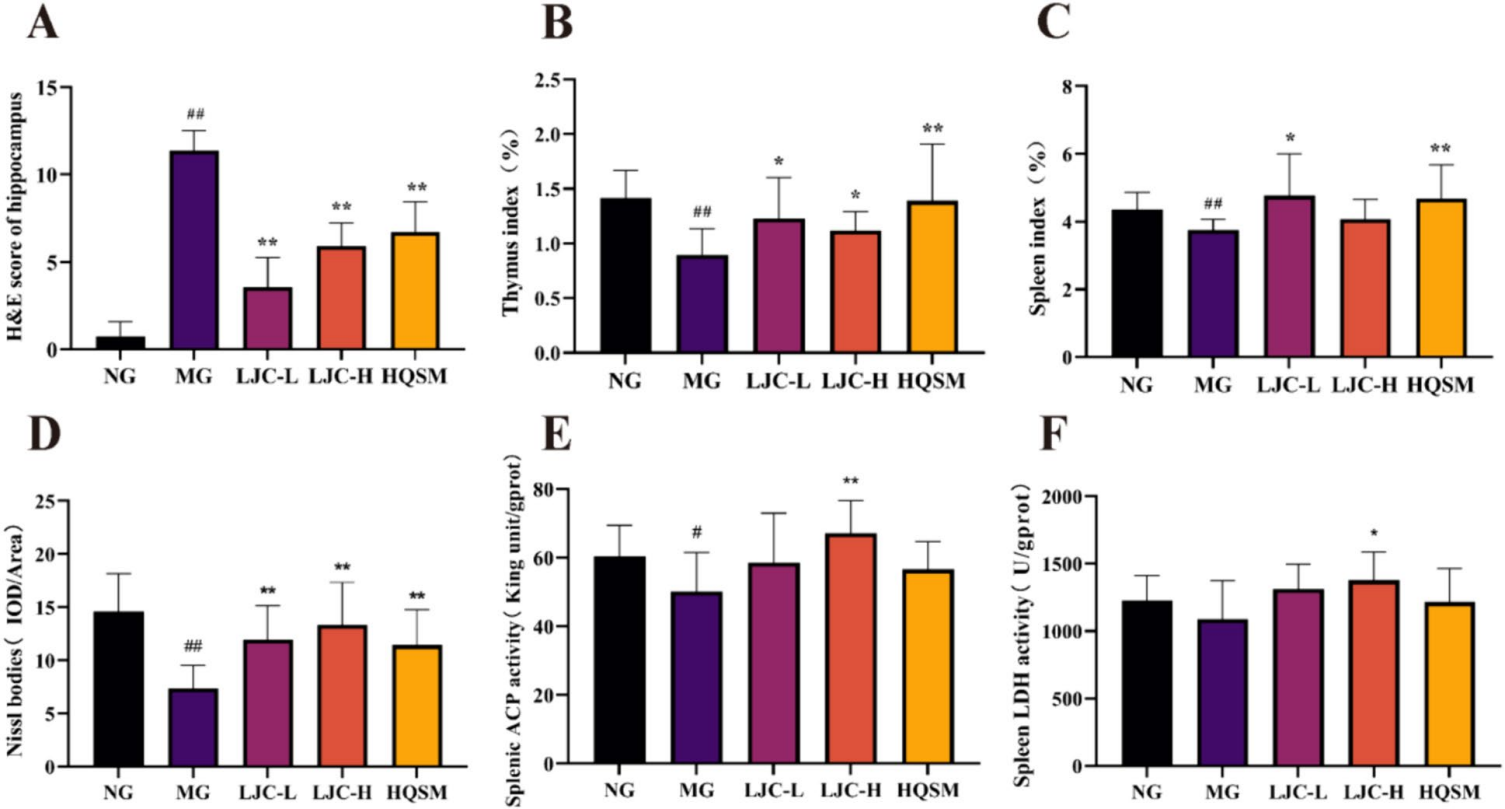
Quantitative histopathological evaluation of brain tissue, thymus and spleen. **A** H&E score of hippocampus; **B** Thymus index; **C** Spleen index; **D** Nissl bodies; **E** Spleen ACP viability; **F** Activity of LDH in spleen. Data are expressed as the mean ± SD. ^#^*P* < 0.05, ^##^*P* < 0.01 compare with the control group; ^*^P < 0.05 and ^**^P < 0.01 compare with the model group

The results of H&E staining in Figs. 5A and 6A revealed that the hippocampal neurons in the normal group exhibited abundant numbers and a well-organized arrangement, with most of them being round or oval-shaped. In contrast, the model group displayed a reduction in hippocampal neuron count, accompanied by disordered cellular arrangement and irregularly shaped nuclei exhibiting pyknosis and dark staining. We summarized the description of hippocampal damage in the literature to set the scoring rules [65–67]: The degree of cell compact arrangement (0–4 points), the degree of nuclear damage (0–4 points), the degree of cell vacuolization and edema (0–4 points). Twelve subjects were randomly selected to evaluate the hippocampal damage according to the scoring rules. The quantitative analysis results showed that compared with the normal group, the damage in the model group was significant (*P* < 0.01). And compared with the model group, all treatment groups effectively improved the hippocampal injury (*P* < 0.01).

Nissl staining as Fig. 5B showed that the Nissl bodies of normal group mice were abundant, neatly arranged, and darkly stained, most of which were triangular. The Nissl bodies of model group mice were reduced in number, disordered in arrangement, and shallow in color, with pyknosis and different shapes. Compared with the model group, the Nissl bodies of each treatment group increased in number and morphology. The statistical results of Nissl staining in Fig. 6D showed that compared with the normal group, the number of Nissl bodies in the model group was significantly reduced (*P* < 0.01); Compared with the model group, the number of Nissl bodies in each treatment group was significantly increased (*P* < 0.01). These results suggest that the administration of LJC can enhance hippocampal nuclear condensation and augment the quantity of nissellites in the cerebral cortex.

### The effects of LCJ on the immune capacity of thymus and spleen

To evaluate the effect of LJC on immune organs, H&E staining of thymus and spleen and ACP and LDH activities of spleen were performed.

Results as depicted in Figs. 5C** and **6B, the thymic lobules of mice in the normal group exhibited clear boundaries between the medulla and cortex, with densely and regularly arranged abundant lymphocytes within the cortex. Conversely, in the model group, there was an indistinct boundary between the medulla and cortex, accompanied by an enlarged diffusion of medulla and a reduced number of sparsely arranged lymphocytes, and the thymus index was significantly reduced (*P* < 0.01). Following LJC administration, structural damage to the thymus was alleviated in each group as evidenced by a relatively clear boundary between the medulla and cortex, a significant reduction in medullary area, a relatively close arrangement of lymphocytes, and the thymic index was up-regulated in different degrees (*P* < 0.05, 0.01).

We can see from Fig. 5D** and **6C that the spleen structure of the normal group mice appeared histologically intact, with a distinct demarcation between the red pulp and white pulp, and an abundance of densely arranged lymphocytes within the white pulp. In contrast, mice in the model group exhibited compromised spleen structure characterized by an indistinct boundary between the red pulp and white pulp, a loose overall architecture, reduced area of white pulp, decreased number of loosely arranged lymphocytes and significantly reduced spleen index (*P* < 0.01). However, following LJC treatment, there was evident amelioration in spleen structural damage across all groups as indicated by a clear demarcation between the red pulp and white pulp, increased area of white pulp, enhanced lymphocyte population and elevated spleen index (*P* < 0.05).

The results of ACP and LDH in spleen are shown in Fig. 6E, F. Compared with the normal group, the activity of ACP and LDH in spleen of mice in the model group decreased, but the change of LDH activity was not statistically significant. Compared with the model group, LJC-H could significantly enhance the activities (*P* < 0.05, 0.01), suggesting that LJC could restore the pathological damage of spleen and thymus in immunocompromised mice with sleep disorders.

### The effects of LJC on GABA and Glu contents and GABA pathway proteins in mice

**Fig. 7:**
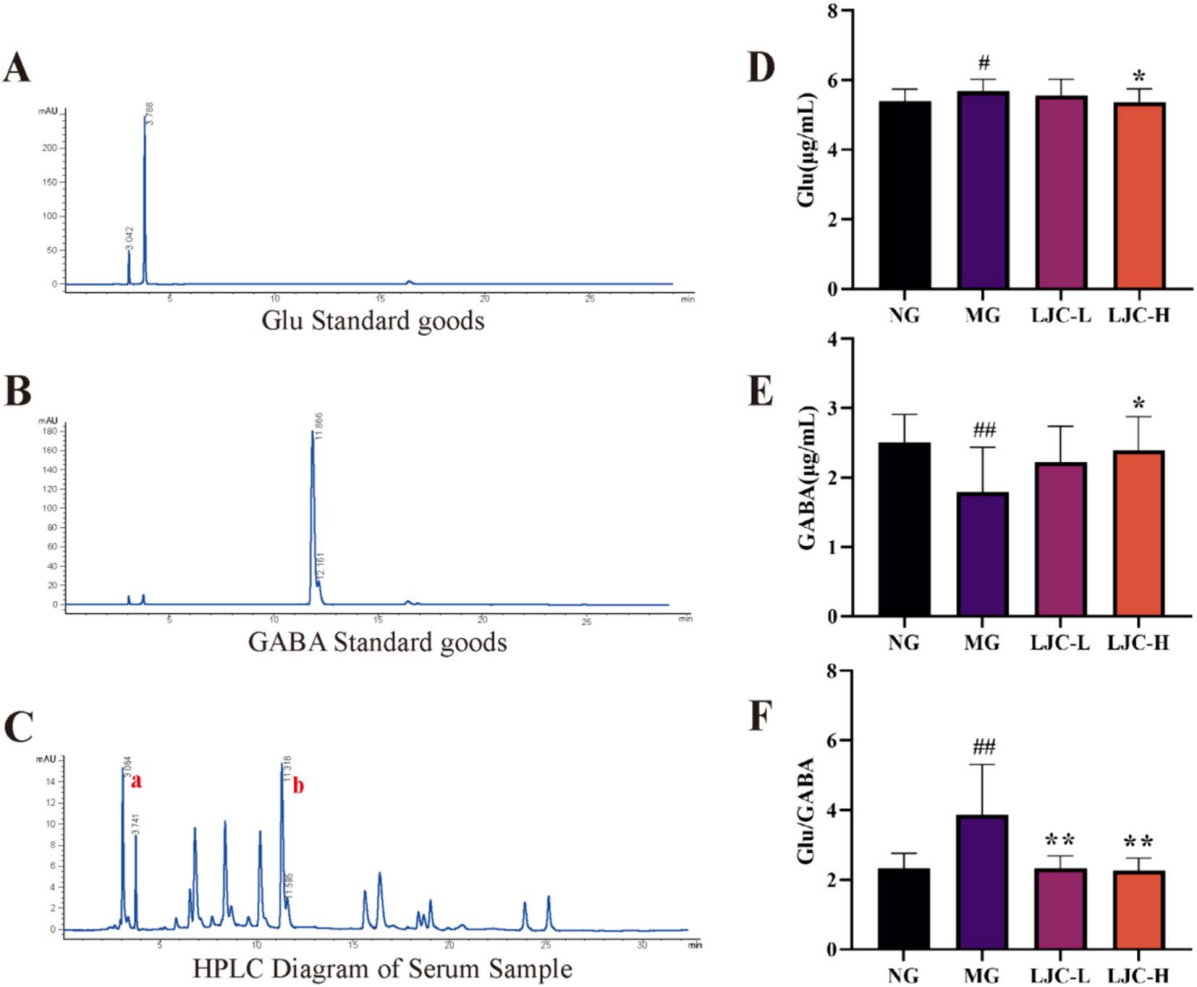
The effects of LJC on GABA and Glu contents. **A** HPLC diagram of Glu standard; **B** HPLC diagram of GABA standard; **C** HPLC representative map of serum samples; **D** Glu content; **E** GABA content; **F** Glu/GABA. Data are expressed as the mean ± SD. ^#^*P* < 0.05, ^##^*P* < 0.01 compare with the control group; ^*^P < 0.05 and ^**^P < 0.01 compare with the model group

The levels of GABA and Glu in the blood of the mice were determined by HPLC. Compared with the normal group, the serum Glu content and Glu/GABA ratio in the model group were significantly increased (*P* < 0.05, 0.01); Compared with the model group, the serum Glu content and Glu/GABA ratio in the LJC group were significantly decreased (*P* < 0.05, 0.01). GABA content was just opposite. This suggests that LJC may affect the sleep state of sleep-deprived mice by regulating Glu and GABA levels (Fig. 7A–F).

**Fig. 8:**
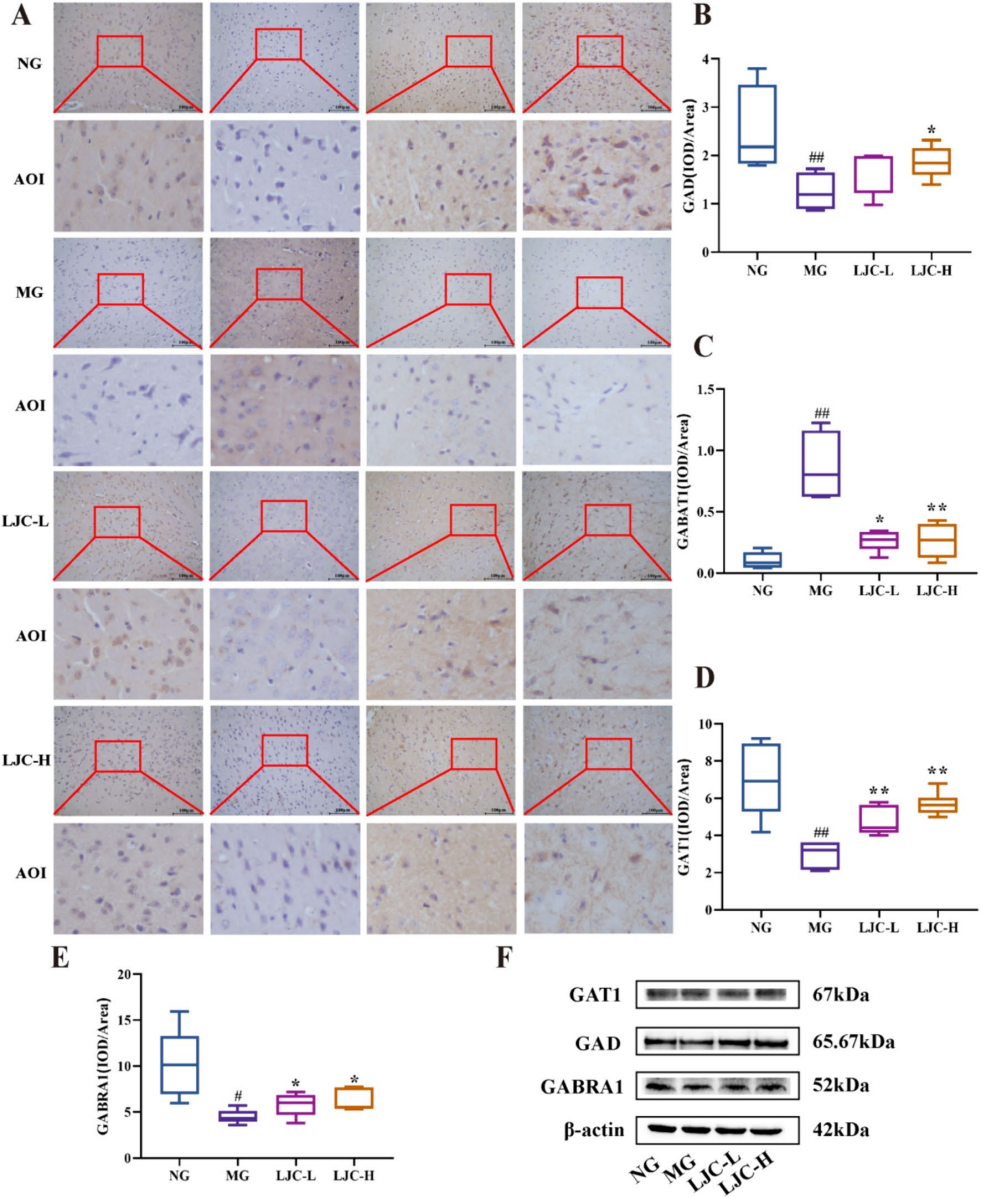
The effects of LJC on GABA pathway proteins in brain tissuecretion. **A** Immunohistochemical representation of GABA pathway proteins (400 ×); **B** Statistical diagram of GAD protein expression; **C** Statistical map of GABAT1 protein expression; **D** Statistical diagram of GAT1 protein expression; **E** Statistical diagram of GABRA1 protein expression; **F** Representative diagrams of GABA pathway protein expression. Data are expressed as the mean ± SD. ^#^*P* < 0.05, ^##^*P* < 0.01 compare with the control group; ^*^P < 0.05 and ^**^P < 0.01 compare with the model group

To evaluate the effect of LJC on GABA pathways, we examined the protein expression of GABRA1, GAT1, and GAD in brain tissue. As shown in Fig. 8A–E, immunohistochemistry results showed that compared with the normal group, the expression of GABRA1, GAT1, and GAD protein in the model group was significantly decreased (*P* < 0.01). After LJC administration, all the indexes showed varying degrees of increase compared to the model group. Conversely, the expression of GABAT1 protein exhibited an opposite trend. As shown in Table 2 and Fig. 8F, the WB results showed that compared with the normal group, the protein expression of GABRA1, GAT1 and GAD in the model group was decreased, and the contents of these three proteins were increased to varying degrees after LJC administration.

**Table 2:** WB results of GABA pathway protein expression

Group	GABRA1	GAD	GAT1
NG	0.509 ± 0.045	0.906 ± 0.020	0.889 ± 0.075
MG	0.352 ± 0.075^#^	0.761 ± 0.051	0.824 ± 0.082
LJC-L	0.503 ± 0.191	0.889 ± 0.015	0.843 ± 0.031
LJC-H	0.658 ± 0.189^*^	0.992 ± 0.024^*^	0.923 ± 0.105

### ***The expression of CD***^***4***+^***and CD***^***8***+^***proteins***

**Fig. 9:**
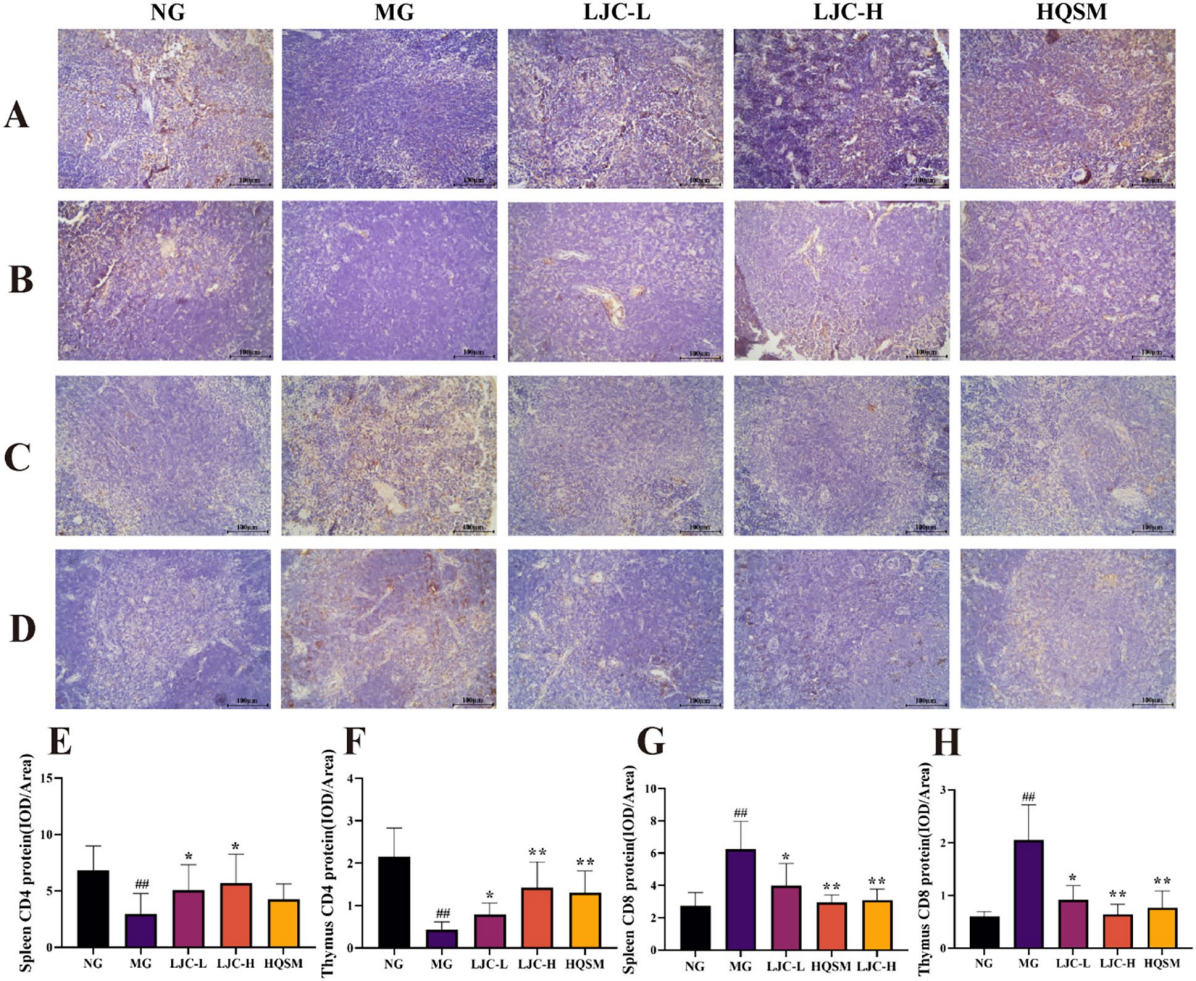
The expression of CD^4+^ and CD^8+^ proteins. **A** Representative diagram of splenic CD^4+^ immunohistochemistry (400 ×); **B** thymic CD^4+^ immunohistochemical representative plot (400 ×); **C** CD^8+^ immunohistochemical representative diagram of spleen (400 ×); **D** thymic CD^8+^ immunohistochemical representation (40 ×); **E** Statistical graph of splenic CD^4+^ expression; **F** Statistical diagram of thymic CD^4+^ expression; **G** Statistical diagram of splenic CD^8+^ expression; **H** Statistical plot of thymic CD^8+^ expression. Data are expressed as the mean ± SD. ^#^*P* < 0.05, ^##^*P* < 0.01 compare with control group; ^*^P < 0.05 and ^**^P < 0.01 compare with model group

The CD^4+^ content of the spleen and thymus in the model group showed a significant decrease compared to the normal group, as depicted in Fig. 9 (*P* < 0.01). Conversely, administration of LJC significantly increased CD^4+^ cell count when compared to the model group (*P* < 0.05, 0.01). However, there was an opposite trend observed for CD^8+^ content.

## Discussion

The primary function of the immune system is to monitor and interpret external incursions and potential threats, subsequently initiating appropriate defense responses. Simultaneously, it monitors the status of internal organs, enhances resistance against disturbances, and maintains homeostasis. Disruption of the body’s immune equilibrium can affect sleep quality [68]. The act of sleeping is a crucial physiological function. However, owing to the accelerated pace of life and the mounting social pressure, an increasing number of people are afflicted by sleep disorders [69]. Such disorders are commonly observed in various diseases and are believed to disrupt the physiological processes regulating the immune system, thereby leading to the development of diseases [25, 71, 79]. In our study, the bad living habits of people in today’s society, such as sleep disorders, poor diet, and overwork, were simulated. The results showed that the mice exhibited a phenomenon of “consumption” and displayed symptoms consistent with “deficiency syndrome,” which were effectively ameliorated by LJC administration.

Autonomic activity experiments, elevated plus maze tests, and open-field tests are commonly employed to assess the sedative, anxiolytic, or stimulating effects of drugs on model mice [72]. Compared with the model group, LJC significantly reduced the motor activity of sleep-deprived mice, prolonged the duration of pentobarbital (50 mg/kg)-induced sleep, and exerted a good sedative and restaging effect.

Several studies have established that the seemingly autonomous immune and nervous systems mutually monitor, interpret, and regulate each other. The nervous system actively monitors, interprets, and regulates the behavior of immune cells and their intricate functions, whereas neurons and glial cells in the nervous system are subject to immune surveillance and their physiological function even relies on factors derived from immune cells [67]. The neuro–immune units (NIU) serve as the fundamental structural entities for neuro–immune interactions within tissues and organs [73]. NIU refers to the colocalization of neuronal processes and immune cells in specific anatomical regions of the body. NIU also denotes the association of neuropeptides, neurotransmitters, cytokines, and other effector molecules for information transmission, which ultimately forms a bidirectional functional interaction unit. NIUs are distributed across various tissues, including the bone marrow, thymus, spleen, lung, skin, intestine, and brain. The nervous system interacts with immune cells either directly or indirectly via neurotransmitters and neuroregulatory factors. Immune cells sense alterations in neurotransmitter levels of the local tissue environment via autocrine or paracrine signaling mechanisms, thus regulating the body’s immune response [74–79].

The hippocampus is the brain region that is most vulnerable to the influence of stress and other pathological conditions [80]. Investigations have proved that the effects of sleep deprivation on the hippocampus are deterministic [81, 82]. Recently, Zhao [83] et al. used amide proton transfer weighted (APTw) imaging to detect disordered hippocampal protein suppression in sleep-deprived rats. Nissl staining revealed that hippocampal APTw signals were positively correlated with the number of surviving neurons. In this study, the hippocampus of model mice showed signs of damage, such as reduced and disorganized neuronal population as well as a decrease in the number of nissl bodies. LJC was significantly efficacious in ameliorating hippocampal damage and promoting an increase in nissl body count.

GABA/Glu, a pair of inhibitory/excitatory neurotransmitters that act on neurons and are present extensively in the central nervous system, play a role in sleep regulation [84]. Patients with sleep disorders often display decreased levels of GABA, which may be accompanied by decreased GAD, GATs, and GABRA1 levels and increased GABAT levels [85, 86]. The findings indicated that LJC upregulated the serum GABA content, downregulated the ratio of Glu content to Glu/GABA, increased the expressions of GABRA1, GAT1, and GAD, and decreased the expression of GABAT1 in the model mice. Thus, sleep disorders can be alleviated.

Studies have observed that both chronic and acute sleep deprivation can alter the functioning of the immune system, including changes in organs such as the thymus and the levels of cells and factors such as lymphocytes, neutrophils, natural killer cells, and IL-6 [13, 22, 87, 88]. Being the major lymphoid organ, the thymus is a pertinent site for the development, differentiation, and maturation of T lymphocytes [89]. The spleen is the largest secondary lymphoid organ in the body, where several T and B lymphocytes settle down and participate in various immune functions [90, 91]. Wu [92] et al. documented that traditional Chinese medicine increased the activities of acid phosphatase (ACP) and lactate dehydrogenase (LDH) in the spleen of immunocompromised mice. These enzymes are key markers of macrophage activation [93, 94]. Pathological alterations and related indicators in the thymus and spleen were analyzed. The results showed that the structures of these organs were damaged in the model group and that the activities of spleen ACP and LDH were decreased. However, LJC significantly alleviated the damage to these organs and reversed the decrease in spleen ACP and LDH activities.

B cell and T cell activation can be used as a general indicator of specific immune activation [95]. When stimulated by an antigen, B cells transform into plasma cells and secrete antibodies to perform their functions [96–98]. The level of hemolysin can reflect the proliferation and differentiation abilities of B cells [99–101]. The results from this study demonstrated that the levels of serum hemolysin and serum immunoglobulin IgA, IgG, and IgM were significantly decreased in the model group and that LJC could significantly upregulate the levels and augment the immune function.

As the center of the immune system, the levels of CD^4+^ and CD^8+^ T lymphocytes can indicate the condition of the body’s immune system. When the ratio of CD^4+^ and CD8^+^ T lymphocytes decreases, it indicates that functions related to cellular immunity are inhibited [102, 103]. Furthermore, the findings alluded that compared with the model group, LJC significantly increased the CD^4+^ content, decreased the CD^8+^ content, and increased the CD^4+^/CD^8+^ ratio. Delayed-type hypersensitivity is an immune response mediated by CD^4+^ T cells, which can reflect the cellular immune function of mice by measuring the degree of foot swelling after 24 h [104]. The degree of paw swelling was significantly reduced in the model group, whereas in the LJC group, it was significantly increased.

**Fig. 10:**
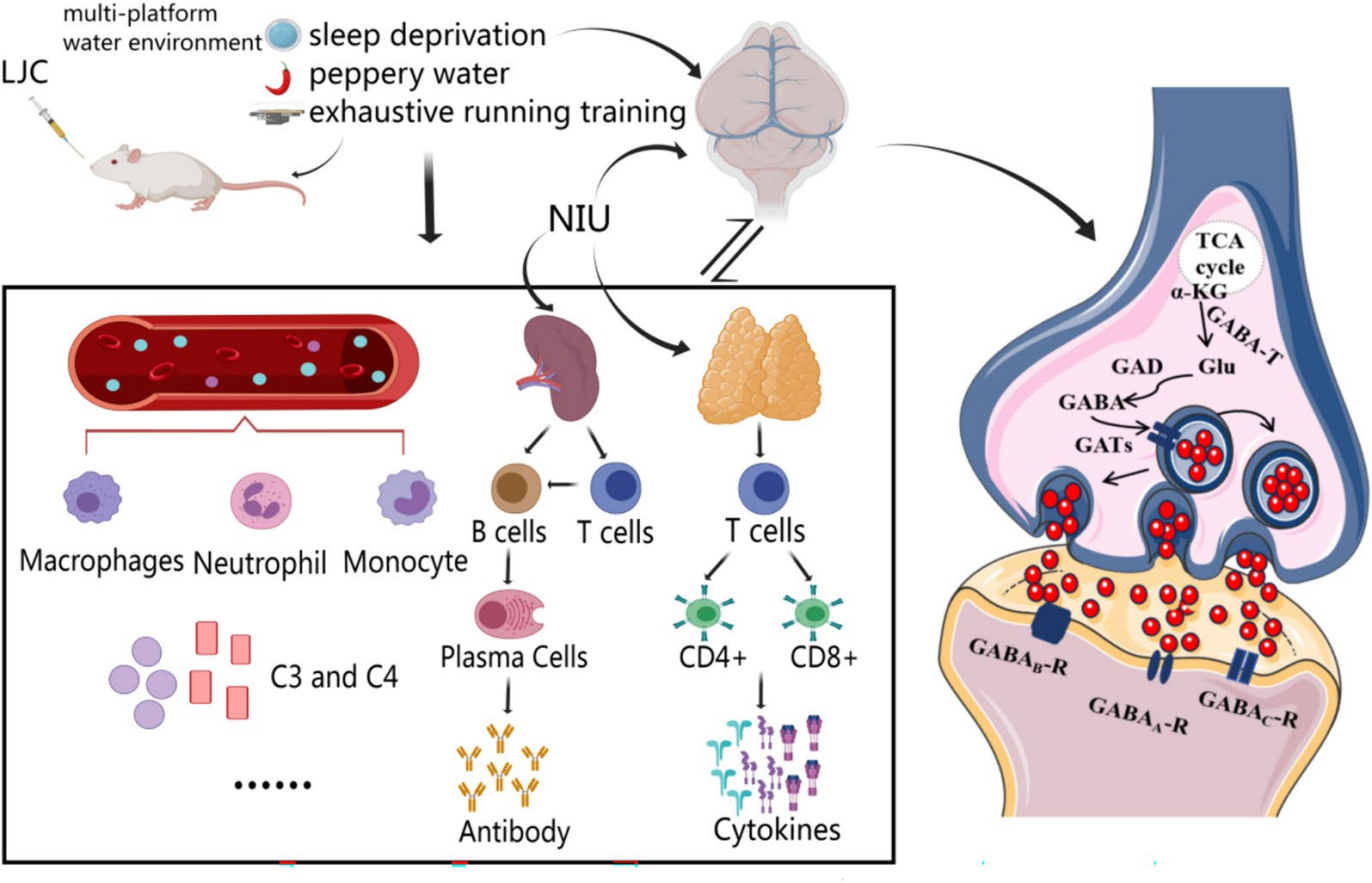
Schematic representation of the mechanism underlying the ameliorative effects of LJC on immunocompromised and sleep-deprived mice through modulation of the GABAergic neuro-immune system

In addition, the complement system is a vital component of the nonspecific immune system [105, 106]. Complement mediators, particularly C3a, can activate neutrophils, mast cells, monocytes/macrophages, T cells, B cells, etc. [107–109]. The complement system is activated predominantly via three pathways—classical, lectin, and alternative—which converge in C3 activation. C4 is involved in the classical and lectin complement pathways [110]. Previous studies have reported that the levels of serum complement C3 and C4 are lower in immunocompromised individuals than in healthy people [111, 112]. Similar results were obtained in the present study too. The levels of complement C3 and C4 were significantly decreased in the serum of the model mice, and those in each dose group of LJC were significantly increased. The number of white blood cells and lymphocytes was significantly decreased and that of central granulocytes was significantly increased. LJC administration, however, significantly increased the number of white blood cells and lymphocytes and decreased the number of neutrophils (Fig. 10).

## Conclusion

This study found that LJC can regulate the content of GABA/Glu and the expression level of GABA metabolic pathway-related proteins in the brain of mice, enhance the specific and non-specific immune function of model mice, and thus improve the state of low immunity and sleep disorders in model mice. Therefore, it is possible to consider developing LJC as a new drug or health food to cope with the increasing number of sub-healthy people with low immunity and sleep problems.

## Data Availability

All data associated with this study are present in the paper. Any information for this study is available by contacting the corresponding authors upon reasonable request.

## Abbreviations

LJC: Modified Qiyuan Paste
HQSM: Huangqi Shengmai Drink
GABA: γ-Aminobutyric acid
Glu: Glutamate
GAD: Glutamic acid decarboxylase
GABAT: γ-Aminobutyric acid transaminase
CATs: GABA transporters
GABRA1: GABAA receptor α1 subunit
EPM: Elevated plus maze
OFT: Open Field test
DTH: Delayed hypersensitivity
EDTA: Ethylene diamine tetraacetie acid
ELISA: Enzyme-linked immunosorbent assay
RIPA: Radioimmunoprecipitation
PVDF: Polyvinylidene fluoride
